# Benzophenones and synthetic progestin in wastewater and sediment from farms, WWTPs and receiving surface water: distribution, sources, and ecological risks†

**DOI:** 10.1039/d1ra05333g

**Published:** 2021-09-27

**Authors:** Siqi Wang, Zhuhao Huo, Jianzhong Gu, Gang Xu

**Affiliations:** School of Environmental and Chemical Engineering, Shanghai University Shanghai 200444 P. R. China xugang@shu.edu.cn; Institute of Applied Radiation of Shanghai, Shanghai University Shanghai 200444 P. R. China; Key Laboratory of Organic Compound Pollution Control Engineering, Ministry of Education Shanghai 200444 P. R. China

## Abstract

Farms and wastewater treatment plants (WWTPs) are important sources of endocrine disruptors, which may have potential adverse effects on the nearby receiving river and potential human health risks. Benzophenone (BPs) and synthetic progestin were determined in water and sediment samples of the discharge source and receiving river. BPs and synthetic progestin ranged from not detected (N.D.) to 400.53 ng L^−1^ in water samples and from N.D. to 359.92 ng g^−1^ dw in sediment, respectively, and benzophenone-3 (BP-3) and ethinyl estradiol (EE2) were the main detected objects. Correlation analysis showed that pollutants discharged from livestock farms were the main contributor to the receiving river. The distribution of pollutants in different regions was related to higher population density and livestock activities. Predicted no-effect concentrations (PNECs) were investigated for ecological risk assessment in the study area, and 86% of the samples exceeded the baseline value of chronic toxicity. Benzophenone-1 (BP-1), benzophenone-3 (BP-3), 4-hydroxybenzophenone (4-OH-BP) and benzophenone (BP) were identified as the main substances that caused medium risk in the aquatic ecosystem. Therefore, BPs and synthetic progesterone should be given more attention in the future.

## Abstract 2

The occurrence, source and ecological risk of BPs and synthetic progestin in farms, WWTPs and their receiving river were investigated.

## Graphical abstract

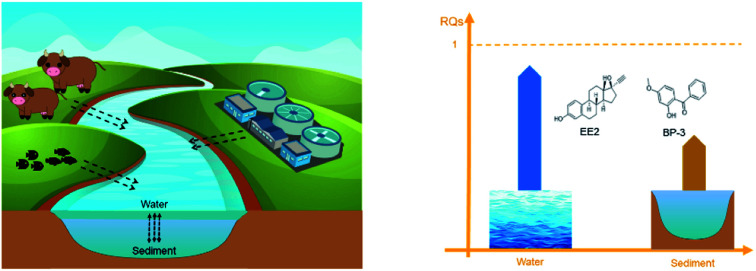

## 1 Introduction

Pharmaceutical and personal care products (PPCPs), as emerging pollutants, are frequently detected in different environmental media and have become an important research field. Benzophenones (BPs) and synthetic progestin are widely used as the ingredients of sunscreen, food packaging and oral contraceptives.^1–3^ The maximum levels of BPs in sunscreens were 10%, 10%, 6% and 5%, respectively, in China, the United States and Japan.^4–6^ South Korea regulates that the maximum allowable amount of 2,2′-dihydroxy-4-methoxy benzophenone in sunscreen was 3%.^7^ BP-3 was also widely used as a UV filter for food plastic packaging, paint and textiles to prevent the color from being photodegraded.^8^ Synthetic progestin is the main active ingredient of female oral contraceptives, which was also applied in animal husbandry to control livestock reproduction.^9^ According to reports from European countries, the consumption of synthetic progestin ranged from 0.34 kg per year to 9864 kg per year.^10^ The consumption of progestin was about 1723 kg per year in the UK, which was far greater than that of estrogen and androgen.^10^ These chemicals have gained growing attention since these compounds have been found to have adverse effects on ecosystems and wildlife.

BPs and synthetic progestin belong to endocrine disrupting chemicals (EDCs), which may interfere with the normal endocrine function of humans and animals even at lower exposure levels.^11^ The adverse effects of BPs and synthetic progestin on aquatic organisms have been reported, specifically, including endocrine disrupting properties, carcinogenicity, reproductive, developmental toxicity and neurotoxicity.^12^ Studies have shown that in the acute toxicity test of zebrafish, the concentration of BP-3 ranging from 2.40 μg L^−1^ to 3.12 μg L^−1^ might lead to hatching reduction and deformity of zebrafish in the early development stage.^13^ Overturf *et al.* exposed female fathead minnows to a concentration of 0.8 ng L^−1^ levonorgestrel (LNG), and found that the structure of female eggs was changed significantly, and 70% of the females lose their ability to spawn.^14^

Livestock farms and aquaculture farms were also important sources of the EDCs substances in the environment. In order to increase the weight and reproduction rate of animals in practical production, farms will give exogenous drugs to animals to meet the production needs. Since hundreds of animals live in the same area, various synthetic progestin were detected in the farms and their surrounding environment. The highest detected concentration of diethylstilbestrol (DES) in surface water near the livestock farm in Taiwan, China reached 12 ng L.^15^ Zhang *et al.* determined 31 kinds of sterol hormones in the river near the cattle farm, with a maximum detected concentration of 11 200 ng L.^16^ BPs and synthetic progestin excreted by humans mainly enter wastewater treatment plants (WWTPs),^3,17^ while these compounds produced by animals mainly appear in animal farms.^18^ However, EDCs were not the main removal targets of urban WWTPs, so a considerable part of endocrine disruptors cannot be effectively degraded.^19^ Therefore, incompletely degraded pollutants and metabolites entered the surface water and sediment with the effluent of WWTPs, which posed a potential threat to the health of aquatic organisms. A variety of BPs and synthetic progestin have been detected in surface water, soil, sediment and WWTPs.^20–22^ The concentrations of BPs detected in rivers and WWTPs in South Korea were 62.9–412 ng L^−1^ and 108–5055 ng L^−1^, respectively.^23^ The 113 kilometer Huangpu River is the most important river in Shanghai, China and also the main receiving water body for Shanghai's wastewater discharge, which is a multi-functional river with the value of drinking water source, shipping, flood drainage, fishery and tourism. With the rapid development of urbanization and industrialization, the water quality of Huangpu River is increasingly deteriorating. Based on the potential risks faced by the ecological community, it is necessary to understand the occurrence, distribution and fate of BPs and synthetic progestin in the receiving environment.

Many investigations have been conducted to understand the occurrence and fate of target chemicals in wastewater and sediments. However, previous studies mainly focus on a single kind of pollution source, such as hospitals, WWTPs.^24^ There is a paucity of information on the contribution pattern of chemicals discharged into the nearby environment through multiple pollution sources. Therefore, a variety of sources, including livestock farms, aquaculture farms and WWTPs, need to be investigated to understand the pathway of substances in aquatic ecosystems. In addition, China is one of the largest consumers of BPs and synthetic progestin in the world, but the water quality criteria of these compounds have not been established to regulate the use and discharge of chemicals. The traditional risk assessment method is carried out by analyzing the potential carcinogenic risk level of the target substance.^25^ Species sensitivity distributions (SSD) method is a more ecological relevant approach when compared to traditional risk assessment methods, which comprehensively accounts for the bioaccumulation and toxicity of each chemical to derive its ecological hazard level.^26^

The aim of this study was to (1) determine the occurrence and distribution of seven BPs and six synthetic progestin in wastewater and sediment of WWPTs, livestock farms and aquaculture farms, and compare target chemicals in China and other developed countries; (2) estimate the contribution of multiple sources of BPs and synthetic progestin discharged into the nearby aquatic environment; (3) evaluate the environmental risks of the target compounds to aquatic species based on the SSD model. The results will provide valuable data for understanding the pollution of BPs and synthetic progestin in rapidly developing cities and the ecological risks brought by these substances.

## 2 Materials and methods

### 2.1 Chemicals and materials

The BPs used in the experiments of this paper include: benzophenone (BP), benzophenone-1 (BP-1), benzophenone-2 (BP-2), benzophenone-3 (BP-3), benzophenone-4 (BP-4), 2,3,4-trihydroxybenzophenone (2,3,4-OH-BP) and 4-hydroxybenzophenone (4-OH-BP). Synthetic progestin include: cyproterone acetate (CPA), gestodene (GES), norethindrone (NTD), diethylstilbestrol (DES), levonorgestrel (LNG) and ethinyl estradiol (EE2). These reagents were purchased from Accu Standard (New Haven, CT, USA). BPA*-*d16 and NTD*-*d8 were purchased from Siaveragea-Aldrich (The Woodlands, Texas, USA). The molecular structures and properties of the target compounds are summarized in Table S1.†

### 2.2 Sample collection

**Fig. 1:**
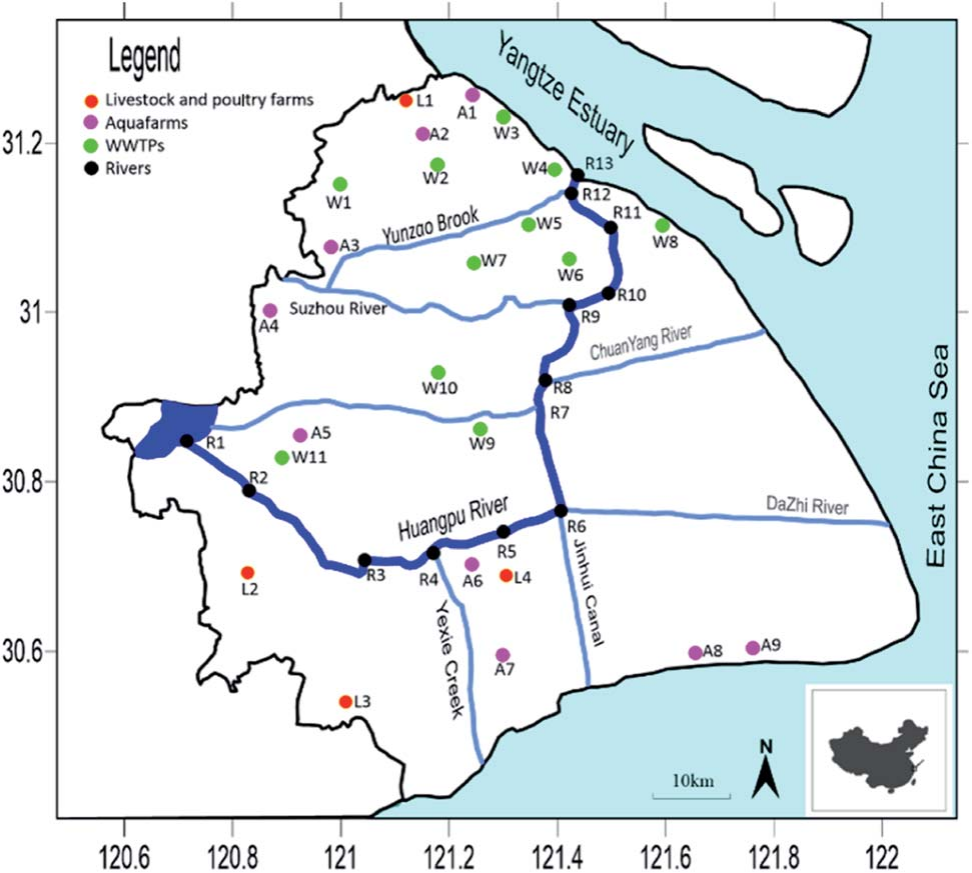
The specific locations of the sampling sites.

The samples were collected in November 2020 respectively. In this study, 13 sampling points in Huangpu River were selected, which were labelled as R1–R13. 5 livestock farms were selected, which were labelled as L1–L5. 9 aquaculture farms were selected, which were labelled as A1–A9. 11 WWTPs were selected, which were labelled as W1–W11. The specific sampling points are shown in Fig. 1 and Table S2.†

Water and sediment samples were collected along the middle of the river cross section. Three replicated water and sediment samples were collected at each site. Water samples were collected at a depth of approximately 50 cm in a tinplate bucket. 1 L water sample at each sampling point were collected, stored in brown glass bottle, pH was adjusted to 3, and stored it at 4 °C after filtering. Sediment samples were collected *via* a stainless steel grab sampler, which was collected in the surficial 10 to 15 cm of bed sediment and stored in polypropylene bags. Thoroughly clean the equipment with deionized water after each use. The sediment samples were freeze-dried at −40 °C, and then stored it in a refrigerator at 4 °C.

### 2.3 Sample preparation and LC-MS/MS analysis

The water samples were pretreated through the solid-phase extraction method (SPE). The water samples were filtered and added 100 μL internal standard. The solid phase extraction column (Oasis HLB, 6 mL, 500 mg) was pretreated with 5 mL methanol and 5 mL ultrapure water, and then the sample passed through the column at a flow rate of 2 mL min^−1^. Then, 5 mL ultrapure water were rinsed, and the column was dried under vacuum for 2 h. The sample was eluted by 3 mL methanol and 3 mL dichloromethane into the nitrogen blowpipe. The eluate was blown dry under mild nitrogen, reconstituted with methanol to 1 mL, filtered through a 0.2 μm filter membrane, and transferred to a sample vial.

Solid samples were pretreated by ultrasonic extraction method. 2 g of freeze-dried solid sample were added 100 ng internal standard and mixed well. Methanol and ethyl acetate (2 : 8, v/v) for extraction, after 15 min of ultrasound, the supernatant was centrifuged at 3500 rpm for 10 min. Then the supernatant was transferred to a 100 mL conical flask. Repeated the above extraction process twice. After the obtained supernatant was combined, the solvent was spin-dried by a rotary evaporator, and then redissolved in 1 mL methanol again. Finally, transferred to a sample injection vial after the filtration through 0.2 μm filter membrane. All the target compounds were analyzed by Agilent 6460 LC-MS/MS.

The LC-MS/MS method has been slightly modified based on previous research.^27^ An Agilent 6460 high performance liquid chromatography-mass spectrometry was used to test the target compounds in samples, and an Agilent Poroshell EC-C18 reverse phase column was employed for separation. Gradient elution of BPs was achieved by mobile phases A (acetonitrile) and mobile phases B (0.1% formic acid). The linear gradient program started at 5% A, increased to 55% A in 5 min, and raised to 80% A in 5 min, maintained for 2 min, then return to 5% A within 3 min, followed by a 3 min equilibration. Mobile phase A was acetonitrile and mobile phase B was 0.1% ammonium acetate solution for synthetic progestin. The gradient program was as follows: 5% A maintained for 5 min, increased to 80% A in 2 min, maintained for 4 min, increased to 85% A in 1 min, maintained for 5 min, and increased to 100% A in 1 min, followed by a 3 min equilibration. Detection was carried out as the negative electrospray ionization mode by multiple-reaction monitoring (MRM). The injection volume was 1 μL. The flow rate was 0.5 mL min^−1^. The drying gas temperature was 400 °C, the sheath gas flow was 13 L min^−1^, the sheath gas temperature was 350 °C, and the nebulizer pressure was 45 psi. The optimization parameters of target compounds are shown in Table S1.†

### 2.4 Quality assurance and quality control (QA/QC)

Each batch of experiments included 6 samples, a spiked matrix and a method blank samples were used to ensure the accuracy of the experimental data. Ten standard solution concentrations (0.1, 0.2, 0.5, 1, 2, 5, 10, 20, 50, 100 ng L^−1^) were used to calculate the calibration curves, and the linear regression coefficients (*r*^2^) of the calibration curves were over 0.99. The limits of detection (LOD) value and the limit of quantification (LOQ) were defined as 3 and 10 times the signal-to-noise (S/N) level of the chromatogram in blank samples, respectively. For chemicals with concentrations below the LOQ, a value of 1/2 LOQ was assigned for analysis. The LOQ of target chemicals ranged between 0.004 ng L^−1^ and 0.03 ng L^−1^ for water samples and between 0.006 ng g^−1^ and 0.1 ng g^−1^ for sediment samples. Details of the LOD and LOQ for each compound are listed in Table S1.† The recoveries of BPA-d16 and NTD-d8 were, respectively, 74.3% ± 21.1% and 86.2% ± 15.1%. The recovery rate of BPs was between 68.5–134%. The recovery rate of synthetic progestin was between 71.3–121.5%. Table S3† are shown the details.

### 2.5 Environmental risk assessment

ETX2.0 risk assessment software was used to establish the species sensitivity distribution (SSD) model. ETX2.0 was a professional SSD software developed by the Netherlands Institute of Public Health & Environment and it was also an official risk assessment software in the European Union.^26^

The predicted no effect concentration (PNEC) value was calculated according to:^28,29^

1 PNEC = HC_5_/AF

where AF is additional safety factor and the value of AF is adopted as 3 for acute benchmark value; AF is adopted as 1 for chronic benchmark value; HC_5_ is the hazardous concentrations for 5% of the species calculated by SSD model.

Risk quotients (RQs) was further used to calculate the health risks of BPs and synthetic progestin exposure:^30^

2 RQs = MEC/PNEC

where MEC is the measured environmental concentration; PNEC is the predicted no effect concentration calculated above.

## 3 Results and discussion

### 3.1 Concentrations and composition of BPs and synthetic progestin in the water

**Fig. 2:**
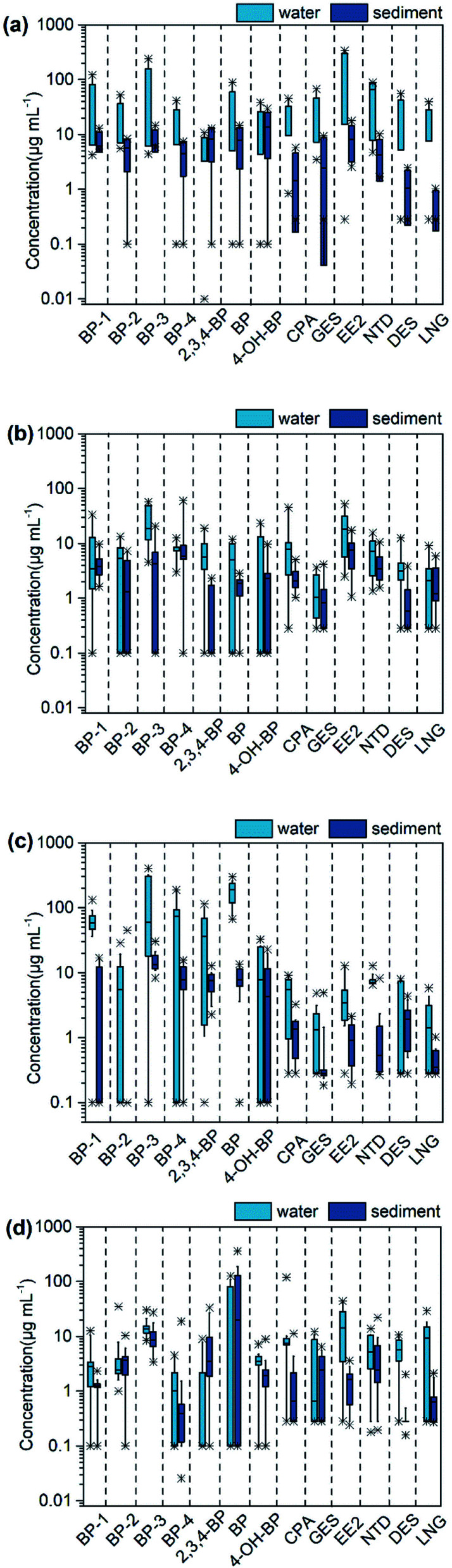
The concentration distribution of target compounds in water and sediment of livestock farms (a), aquaculture farms (b), WWTPs (c), receiving river (d).

The concentrations of BPs and synthetic progestin in livestock farms, aquaculture farms, WWTPs and Huangpu River are shown in Fig. 2 and Table S4.† The detection range of BPs was N.D.–400.53 ng L^−1^, and the average concentration was between 1.68 and 18.01 ng L^−1^. BP-3 has the highest detection rate (95%), followed by BP-1 (84%). The detection range of synthetic progestin was N.D.–338.24 ng L^−1^, and the average concentration was 2.06–7.59 ng L^−1^. NGT has the highest detection rate (95%), followed by EE2 (87%) and CPA (82%). The distribution of different targets showed obvious regional differences, which depended on the source of wastewater.

The effluent from 5 livestock farms was collected, of which L1 was a dairy farm, L2 and L3 were swine farms, and L4 and L5 were poultry farms. All the target compounds were detected in the samples, but the detection types were slightly different. Higher concentrations of EE2 were found in the effluent of swine farms and poultry farms, but not in the dairy farm. BP was not detected in the effluent of the swine farms, but the substance was detected in the poultry farms. Animal numbers and species could explain the different concentrations. The average concentration in the dairy farm (5.55 ng L^−1^) was lower than that in swine farms (6.45 ng L^−1^) and poultry farms (63.95 ng L^−1^). The dairy farm was equipped with an internal wastewater treatment system, which indicated that some pollutants were degraded in the WWTPs. EE2 and BP-3 were respectively the highest detectable concentrations of BPs and synthetic progestin in the effluent of livestock farms, with the maximum concentrations of 338.24 ng L^−1^ (EE2) and 239.42 ng L^−1^ (BP-3), respectively. The concentration of progestogens detected by Liu *et al.* in the washing water of sow pen in a swine farm was 351.4 ng L^−1^, which was close to the detection level in this study.^31^ In this paper, both influent and effluent water samples from a wastewater treatment station of one dairy farm were collected, 12 target chemicals were detected in the influent water and 8 target chemicals were detected in the effluent. The results showed that some target pollutants had been removed after the processing of the wastewater treatment system.

China is one of the largest aquaculture and export countries. In 2015, China exported about 4.1 million tons of aquatic products to Japan, Southeast Asia, the United States, Korea, and Taiwan.^32^ Among the 9 aquaculture farms studied in this paper, 3 were marine farms (A1, A8 and A9), and 6 were freshwater farms (A2, A3, A4, A5, A6, A7). The pollution level of marine farms was higher than that of freshwater farms. In marine farms, the detection range of BPs was between N.D. and 52.34 ng L^−1^, and the detection range of synthetic progestin was between N.D. and 52.31 ng L^−1^, BP-2 was not detected. In freshwater farms, the detection range of BPs was between N.D. and 56.98 ng L^−1^, and the detection range of synthetic progestin was between N.D. and 41.20 ng L^−1^. BP-3 has the highest average detection concentration (20.11 ng L^−1^), followed by BP-4 (7.03 ng L^−1^), which was similar to the concentration level detected in Guangzhou marine farms (0.19–34 ng L^−1^), but significantly higher than that of marine farms in the Netherlands (<13.70 ng L^−1^) and the Netherlands (6.2–11 ng L^−1^).^33–35^ Compared with freshwater farms, marine farms were mostly carnivorous fish such as *Larimichthys crocea*, *Epinephelus*, Amur catfish, *etc.* Low-fat trash fish is. Low-fat miscellaneous fishes was used in the feed, which contain endogenous steroids. Therefore, appropriate management measures should be taken to control the use of feed, especially in the marine aquaculture environment.

The concentration of BPs in the effluent of the 11 WWTPs was between N.D. and 400.53 ng L^−1^. In this study, BP-3 was the highest concentration of BPs in the effluent from the WWTPs, with an average concentration of 26.51 ng L^−1^, which is similar to reports in New York State, USA (35.6–49.1 ng L^−1^).^5^ The main source of BPs in WWTPs was the domestic wastewater discharged by urban residents. It can be inferred that the utilization rate of BP and BP-3 was higher than other UV filters. BP was classified as a group 2B carcinogen by the International Agency for Research on Cancer (IARC), indicating that this type of substance was carcinogenic to humans.^36^ The mean concentration of synthetic progestin in WWTPs was ranked as NGT > EE2 > CPA > DES > LNG > GES, ranging from N.D. to 12.70 ng L^−1^. The detection frequency of NTD was 100%, followed by EE2 (91%) and CPA (82%). Compared with this study, the detection frequency of NTD in WWTPs in Malaysia was also high, but the detection concentration (188 ng L^−1^) was much higher than in this study.^37^ Synthetic progestin is mainly used in human medicine and animal growth promoters worldwide. Previous studies have shown that the occurrence of synthetic progestin was closely related to population density and was greatly affected by urban activities.^2^

This study collected samples from 13 points of the mainstream of the Huangpu River. The concentration of BPs ranged from N.D. to 127 ng L^−1^, and the average concentration was 1.71 ng L^−1^. The highest concentration and detection frequency was BP, with a detection rate of 100%, and the average concentration was 1.37 ng L^−1^. The detection rates of BP-1, BP-3 and BP-4 were all higher than 80%, and the highest concentrations were 12.60 ng L^−1^, 30.00 ng L^−1^ and 4.50 ng L^−1^, respectively. BP-3 was the most widely used BPs, while the detection rate and detection concentration were not the highest. This might be caused by the easy degradation of BP-3 in the natural environment.^23^ The concentration of synthetic progestin ranged from N.D. to 119.46 ng L^−1^ and the average detected concentration was 3.40 ng L^−1^. The detection rates of EE2 and CPA were both greater than 80%. EE2 was the compound that had the highest detectable concentration in the Huangpu River, with an average concentration of 6.62 ng L^−1^, followed by CPA, with an average concentration of 4.62 ng L^−1^. It is because EE2 and CPA were more stable and have a longer half-life.^37^ Recreational activities, incomplete removal of pollutions in wastewater and other land-based pollution were the main source for their appearance in the environment.^2^ In South Korea, river input from household or industrial discharges might also be the most important source of pollution in Korea Bay.^38^ Several studies have identified BPs in various aquatic media, including wastewater from WWTPs, surface water, sediments, groundwater and drinking water,^39,40^ however, it is still necessary to have a broader understanding of the migration and transformation of such chemicals in the environmental. The research results will provide data support for the implementation of chemical management and wastewater regulations.

### 3.2 Concentrations and composition of BPs and synthetic progestin in sediment

The concentration of BPs in sediment ranged from N.D. to 359.92 ng g^−1^ dw, and the average concentration was between 0.76 and 6.73 ng g^−1^ dw. The concentration of synthetic progestin ranged from N.D. to 21.97 ng g^−1^ dw, and the average concentration was between 0.67 and 2.01 ng g^−1^ dw. The average concentration of BPs followed the order of livestock farm (4.47 ng g^−1^ dw) > WWTPs (2.43 ng g^−1^ dw) > Huangpu River (1.82 ng g^−1^ dw) > aquaculture farm (1.40 ng g^−1^ dw). The average concentration of synthetic progestin followed the order of aquaculture farm (1.88 ng g^−1^ dw) > livestock farm (1.83 ng g^−1^ dw) > Huangpu River (0.95 ng g^−1^ dw) > WWTPs (0.71 ng g^−1^ dw). It can be found that the pollution level of synthetic progestin in aquaculture farms was relatively high, while BPs level was relatively low. The aquaculture farms in this study were traditional pond farms, it can be inferred that the various target compounds detected in the farms were mainly derived from steroid compounds intentionally added in commercial feed. In sediment, BP-3 had the highest detection frequency and concentration among the BPs, and EE2 was the synthetic progestin with the highest concentration. The detection frequency of EE2 and NGT in the sediment of livestock farms and aquaculture farms was 100%. The detection frequency of LNG in the sediment of aquaculture farms was 89%, which was much higher than that in livestock farms (40%). This phenomenon was different from that in water samples, which can be attributed to the adsorption of substances in sludge and sediment.^41^ The detection rates of DES in the sediment of WWTPs, aquaculture farm, livestock farm and Huangpu River were 91%, 67%, 60% and 23%, respectively. BP content in sediment was relatively low, and the average detectable concentrations in WWTPs, Huangpu River, livestock farm and aquaculture farm were 5.45 ng g^−1^ dw, 4.34 ng g^−1^ dw, 3.72 ng g^−1^ dw and 1.31 ng g^−1^ dw, respectively. The concentrations of GES, LNG and BP-2 were all lower than 1 ng g^−1^ dw. Studies have shown that 4-OH-BP may be produced in the degradation process of BP-3 and other BPs, so 4-OH-BP has a higher level in the sediment.^42^ Most UV-filters have a high octanol–water partition coefficient (log *K*_ow_ > 3), showing significant accumulation potential in suspended solids, sediment and organisms.^43^

**Fig. 3:**
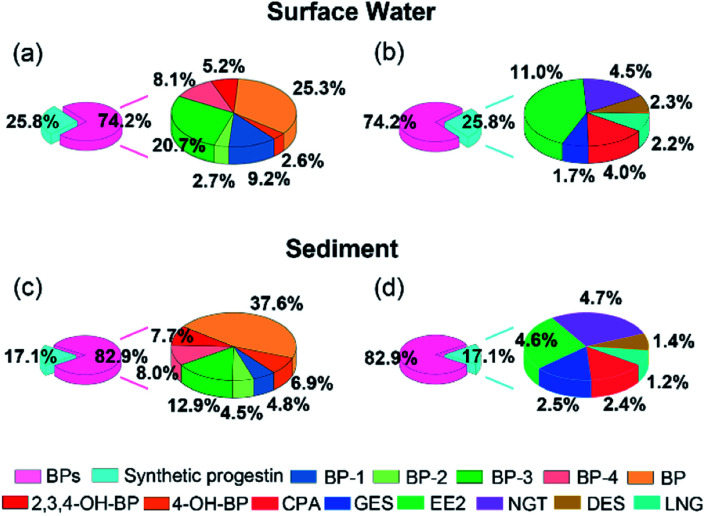
Composition profiles of BP and synthetic progestin in surface water (a) and (b) and sediment (c) and (d).

Fig. 3 showed the contribution of BPs and synthetic progestin in surface water and sediment. BPs and synthetic progestin in water samples accounted for 74.2% and 25.8%, respectively, and their proportion in sediment was 82.9% and 17.1%, respectively. BP-3 and EE2 were the main pollutants detected in water samples, accounting for 20.8% and 11.0%, respectively. The proportion of BP (37.7%) in sediment was higher than that of BP-3 (12.9%), which may be due to compared with BP, BP-3 has good water solubility and it was difficult to be adsorbed by sediments. The higher concentration of EE2 may be related to its higher usage and wide range of use in cities. In the aquaculture farm, the increase of EE2 content indicated that the farms may use hormone containing feed.^44^ EE2 as an animal feed additive has been banned by the European Union and China, but it can still be detected in the feces of livestock and poultry discharged from some livestock farms and aquaculture farms in China. For example, Yao *et al.* detected a high concentration of EDCs with a range of 0.85–2619 ng g^−1^ ww in wild fishes from two major rivers in China;^45^ Kei *et al.* also found a high concentration of EE2 in receiving river of swine-raising facilities.^46^ BP-3 and EE2 as an indicator of contaminant loading should be included in future water monitoring programs to provide information support for the formulation of relevant regulations.

The concentration of EDCs in surface water and sediment varies greatly in different countries (Table 1). For instance, the concentration of EE2 in the surface waters of China and Brazil was less than a dozen ng L^−1^,^52,53^ while in Iran, the concentration of EE2 was as high as 63 ng L.^54^ Among the developed countries in Europe and America, as well as Korea, the detectable concentration of synthetic progestin was lower than that in developing countries.^55–58^ This may be related to geographical location, number of population and animals, and economic development level. Meanwhile, it may also be due to the more perfect and powerful drug monitoring system in developed countries, which makes drug abuse not common as in developing countries. Besides, research on lake water in Asia showed that hormones in lakes were mainly caused by residential, industrial and agricultural, natural hormones dominated the detected hormones, with a total concentration of 630 ng L^−1^, which belong to the same order of magnitude as the detectable concentration in this study.^59^

**Table 1:** Comparison of some target compounds in China and other countries^a^^35,47–51^

Country	Compound	Surface water (ng L^−1^)		Sediment (ng g^−1^ dw)	
		Range	Mean	Range	Mean
**Livestock farms**					
USA	17α-estradiol	—	98	—	—
New Zealand	17α-estradiol	110–11 000	—	—	—
Japan	BP	650–680	—	—	—
China	CPA	2.62	—	—	—
China	NTD	21.60–42.70	—	3.45–321	4.20

**Aquaculture farms**					
China	P4	6.10–3.30	—	0.50–109	—
China	Cortisol	0.55–26	1.32	N.D.–270	—
USA	17β-estradiol	650	—	—	—
Netherlands	Cortisol	3.80–217	—	—	—

**WWTPs**					
China	NTD	—	—	<0.08	—
Swiss	BP-3	0.70–7.80	—	—	—
Norway	BP-3	81–593	293	300–8900	—
France	EE2	4.20–15.50	—	—	—
Korea	17α-estradiol	10.70–25.60	18.93	—	—
Spain	17β-estradiol	11.90–203	—	1.32–128	5.69
Australia	17β-estradiol	1.90–4.20	—	—	—
Italy	Estrone	25–132	—	—	—
France	NTD	13–41	—	—	—

**River**					
USA	BP-3	N.D.–136	—	N.D.–4.30	—
France	BP-3	30–125	—	—	—
Swiss	BP	<2–35	—	—	—
Columbia	EE2	—	—	N.D.–54	—
Chile	EE2	—	—	N.D.–2.96	0.87

a ‘—’ means lack of data.

### 3.3 Relationship and spatial distribution of BPs and synthetic progestin in discharge sources and receiving river

**Fig. 4:**
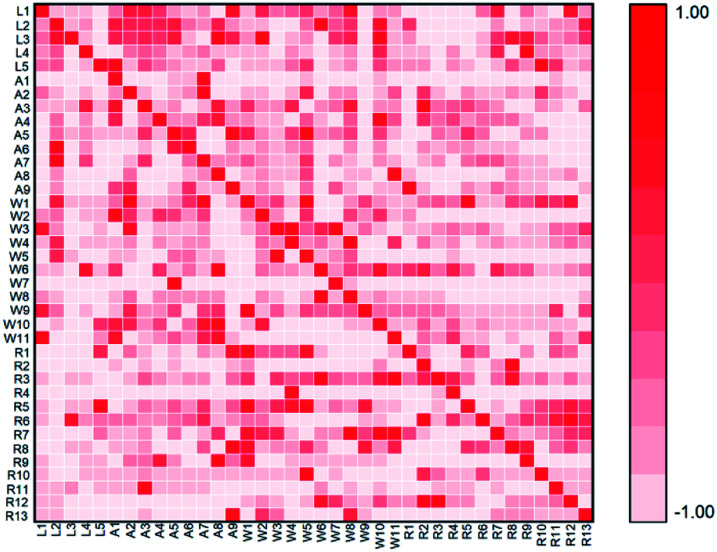
Spearman correlation for effluents of sampling points and receiving river.

In order to study the sources of target pollutants in Huangpu River, correlation analysis was conducted between the effluent from livestock farms, aquaculture farms, WWTPs and Huangpu River, such as (L1, R1), (A2, R1), (W3, R1). The results are shown in Fig. 4. Spearman's correlation analysis was calculated by SPSS19.0 software, and the results were verified by Bartelett *χ*2 test.

By analysing the percentage of each compound in different samples, the correlation between pollutants in the Huangpu River, farms and WWTPs was studied. The correlation between pollutants in the WWTPs and the Huangpu River was relatively weak, which indicated that the WWTPs produced less pollution to the receiving river. However, (R12, W5) showed a significant correlation (Spearman's *r* = 0.691, *p* = 0.128), which may be related to the different treatment systems of the sewage plants. At present, most WWTPs in Shanghai mainly adopt biological treatment methods, including activated sludge process, oxidation ditch process, anaerobic/anoxic/aerobic process (A2/O), membrane bio-reactor (MBR), *etc.* Traditional treatment processes were mainly designed for removing conventional pollutants such as COD, BOD, nitrogen and phosphorus, so there were no targeted treatment methods for some new organic pollutants like steroid hormone compounds.^60^ Previous studies have shown that the removal of steroid hormones by WWTPs might cause a negative growth, the concentration of such substances in the effluent was higher than that in the influent.^43^ For example, in a WWTPs in California, USA, the detectable concentration of E1 in the effluent (38 ng L^−1^) was higher than its concentration in the influent (31 ng L^−1^), which might be due to the dissociation of the conjugated state or the conversion of similar structure compounds under the action of microorganisms.^61^

Intensive livestock farms polluted surface water mainly through the following two ways: one was the direct discharge of sewage and flushing water, and the other was the process of livestock manure stacking in the open air.^62^ In the second case, the pollutants in the manure enter the soil and groundwater in the precipitation process, causing water pollution. In this study, (L1, R1) (Spearman's *r* = 0.841, *p* = 0.036) and (L4, R12) (Spearman's *r* = 0.928, *p* = 0.008) were significantly correlated, which indicated that the organic pollutants discharged from poultry farms were one of the sources of BPs in Huangpu River. There was a low correlation between cattle farms and swine farms. The sewage from cattle farms and swine farms was treated by internal WWTPs before entering the receiving river, which showed that the wastewater treatment system removed the target pollutants to a certain extent.

In aquaculture farms, (A1, R1) (Spearman's *r* = 0.531, *p* = 0.076) and (A4, R6) (Spearman's *r* = 0.438, *p* = 0.009) were significantly correlated, indicating that trace organic pollutants discharged from aquaculture farms might caused pollution to nearby receiving water. In the process of aquaculture, fish excreted hormones and their metabolites directly into the water through gills or bile. Steroids used to prevent or treat fish diseases and promote growth were directly added to the aquaculture water or feed, which was also one of the ways for the hormones to enter the water environment.^63^ Due to the small scale of the aquaculture farms in this study, none of them were equipped with sewage treatment systems. In this situation, the wastewater directly entered into the surrounding river, soil and groundwater, resulting in a relatively high correlation with pollutants in receiving rivers. The results indicated that it is necessary to establish management practices for synthetic progestin emissions under the conditions of intensive livestock farms, and their migration and transformation effects in different media should be considered.

**Fig. 5:**
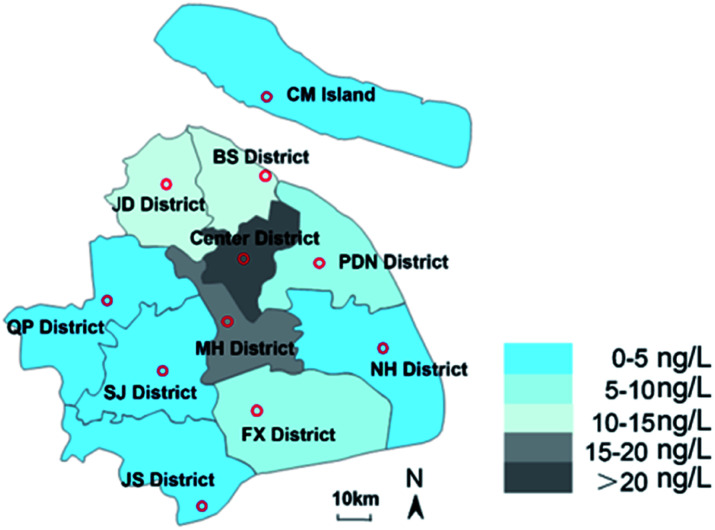
The concentration range of target compounds in each region of Shanghai.

This paper analysed the distribution of pollutants in different administrative districts of Shanghai, and the results are shown in Fig. 5. It can be seen that the average concentration of JD district, BS district, PDN district and FX district was higher than that in other urban districts, which might be related to the dense distribution of livestock farms in this area. The average concentration in Center district was 222.40 ng L^−1^, which might be caused by the higher population density in these districts. The concentrations in NH district, QP district, SJ district and JS district were relatively low, with the average detectable concentration ranging from 0 ng L^−1^ to 5 ng L^−1^. It should be noted that the population density in these districts was also lower than that in the central urban districts. The view that population and discharge flow drives the PPCPs concentration in environment is also supported by Y. Zhu whose recent manuscript ranked the key drivers affecting surface water concentrations.^39^ All 13 target pollutants were detected in MH district, where the breeding industry was concentrated, and the detectable concentrations were much higher than those in the receiving river near the WWTPs (1.2–55.3 ng L^−1^), indicating that the sewage discharged by the farms might cause more pollution to the receiving environment than WWTPs. Some previous studies have also reported the pollution of surface water caused by hormones in livestock manure. The pollution level of Huangpu River was on the rise from upstream to downstream. The pollution levels of R8 and R9 were more serious, which may related to the location closer to the WWTPs. In addition, other tributaries merged into Huangpu River from these two points. R7 and R8 were closed to the city center, and the concentration and detection frequency of these two points increased rapidly. Previous toxicological studies have shown that hormonal, like EE2, with a concentration of less than 10 ng L^−1^ would damage the reproductive ability of fish and decrease its population size.^64^ Considering the concentration of pollutants detected in downstream waters, synthetic progestin may pose a certain risk to aquatic organisms. Due to the continuous expansion of urbanization, agricultural and medical applications, the consumption of EDCs is increasing, and the production of new EDCs is also continuing. This research indicated that BP-3 and EE2 should be considered priority EDCs in Shanghai, China. Future research is required to pay more attention to the persistence and bioaccumulation of these chemicals and their migration and transformation in the environment. This study has confirmed that livestock farms were the main contributor to the receiving river. Therefore, long-term monitoring of the potential source of benzophenones and synthetic progestin in the environment is necessary to be more accurately assess ecological risks.

### 3.4 Environmental risks of BPs and synthetic progestin

Species sensitivity distributions (SSD) method were used to derive the water quality criteria of endocrine disruptors in Huangpu River. The SSD method has been used in ecological risk assessment and water quality criteria derivation by more and more countries and regions.^26^ Compared with the traditional risk assessment methods, SSD method is a statistical method with higher confidence.^65^ SSD curve was constructed using the data of species' acute toxicity and chronic toxicity. The maximum allowable concentration for 95% of species not affected was calculated, which is an important basis for the formulation of environmental safety baseline and the implementation of ecological risk assessment.

The toxicity data of the acute toxicity terminus (LC_50_ and EC_50_) and the chronic toxicity terminus (LOEC and NOEC) of the aquatic organism were originally from the ECOTOX database (http://cfpob.epa.gov/ecotox) (USEPA) (Table S5†). The results of Shapiro–Wilk test showed that the toxicity value of different species was displayed normal distribution (*p* < 0.01). ETX2.0 software was used to establish the SSD model, and the hazardous concentration (HC_5_) value was obtained. The predicted no effect concentration (PNEC) was adopted as the water quality standard to assess the ecological risk in Huangpu River. The PNEC values are shown in Table 2.

The PNEC values of BPs and synthetic progestin^a^ (mg L^−1^)

**Table:** 

	BP-1	BP-2	BP-3	BP-4	4-OH-BP	2,3,4-OH-BP	BP
Acute	157.41	2.27	3.32	1167.23	1067.60	—	628.17
Chronic	171.02	14.57	5.44	13.92	—	—	783.71

a ‘—’ means lack of data, ‘acute’ means acute toxicity standard value and ‘chronic’ means chronic toxicity standard value.

**Table:** 

	CPA	GES	NTD	DES	LNG	EE2
Acute	8.53	—	—	—	—	—
Chronic	1.39	—	0.11	—	0.043	0.001

At present, the relevant water quality standards issued by the governments at home and abroad are mostly aimed at heavy metals, traditional organic pollutants, *etc.*, and there is a lack of new organic pollutants, especially water quality standards for environmental endocrine disruptors. By comparing the PNEC value with the concentration of target compounds detected from the practical samples, it can be observed that the concentration of target substances in all water samples in this study was lower than the acute toxicity standard value, but the concentration of the target substances in 86% exceeded the standard value of chronic toxicity. The results indicated that the existence of such pollutants in Shanghai has chronic toxicity to the reproduction of aquatic organisms, and the occurrence of such pollutants in the environment should be monitored for a long time.

RQs was used to calculate the non-carcinogenic risks faced by Shanghai residents. When RQs < 0.1, it indicates that no adverse reactions to the population; when 0.1 < RQs < 1, it indicates that the population has low adverse reactions to the target pollutant, but there is still a potential risk; when 1.0 < RQs < 10, it indicates that moderate risk faced by the population; when RQs > 10, it indicates that the population has a higher health risk. In this study, the RQs of BP-1, BP-2, BP-3, BP-4, 4-OH-BP, 2,3,4-OH-BP and BP were 0.57, 0.26, 0.84, 0.41, 0.52, 0.37, and 0.83, respectively; the RQs of CPA, GES, NTD, DES, LNG and EE2 were 0.23, 0.05, 0.34, 0.12, 0.21 and 0.35, respectively. It can be seen that the RQs of BP-1, BP-3, 4-OH-BP and BP were above 0.5, which means that the population in Shanghai was facing a moderate risk caused by BPs. Taking the increasing market demand for such substances into consideration, long-term and continuous monitoring of the occurrence levels of target pollutants in different environmental media in Shanghai should be carried out.

## 4 Conclusions

In this work, we studied the occurrence of BPs and synthetic progestin in the wastewater and sediments from farms and WWTPs, and their influence on the nearby rivers and the ecological risk were also investigated. Among the three different kinds of sources, livestock farms had the highest contribution of target compounds in the nearby river, followed by the WWTPs and aquaculture farms. BP-3 and EE2 were screened as priority substances of BPs and synthetic progestin pollutants in Shanghai, China. The SSD model was established to determine PNEC of BPs and synthetic progestin in surface water of Shanghai. The results showed that the PNEC for target compounds was between 0.001 mg L^−1^ and 1167.23 mg L^−1^, and the RQs was ranged from 0.05 to 0.84. 86% of the water samples exceeded the chronic toxicity of PNEC value, indicating a potential ecological risk to aquatic ecosystems. Population density and medical activities are possible determinants that affect the pollution concentration in the aquatic environment. This work systematically reflected the occurrence and source of BPs and synthetic progestin in Shanghai, and the results can provide references for ecological risk assessment and regulatory decisions.

## Author contributions

Siqi Wang: conceptualization, methodology, sample collection, writing original draft preparation. Zhuhao Huo: sample collection. Jianzhong Gu: methodology, funding acquisition. Gang Xu: conceptualization, funding acquisition, supervision, reviewing and editing.

## Conflicts of interest

There are no conflicts to declare.

## Supplementary Material

RA-011-D1RA05333G-s001
