# Supplementary material for: Benzophenones and synthetic progestin in wastewater and sediment from farms, WWTPs and receiving surface water: distribution, sources, and ecological risks
Source: RSC Adv. 2021 Sep 27;11(50):31766–75. doi: 10.1039/d1ra05333g (PMC9041579; doi:10.1039/d1ra05333g)
Supplement: RA-011-D1RA05333G-s001 [file RA-011-D1RA05333G-s001.pdf]

Electronic Supplementary Material (ESI) for RSC Advances.
This journal is © The Royal Society of Chemistry 2021

Table S1. CAS number of the target substance; LogKow, Fragmentor voltage (V), Collision energies (eV), MRM transitions for the quantifier ions, LOD (ng/mL) and LOQ (ng/mL) of each substances

Target substance	CAS NO.	LogKow	Fragmentor voltage	Collision Energy	Polarity	MRM	LOD	LOQ
Benzophenone-1 (BP-1)	131-56-6	2.96	108	19/18	Positive	215.1＞137<br>215.1＞105	0.06	0.02
Benzophenone-2 (BP-2)	131-55-5	2.78	112	16/35	Positive	247.2＞137<br>247.2＞81.2	0.06	0.02
Benzophenone-3 (BP-3)	131-57-7	3.79	93	18/18	Positive	229.1＞151<br>229.1＞105	0.06	0.02
Benzophenone-4 (BP-4)	4065-45-6	0.37	128	18/35	Positive	309.2＞231<br>309.2＞135	0.06	0.02
2, 3, 4-Trihydroxybenzophenone（2,3 ,4-OH-BP）	1143-72-2	-	124	18/28	Negative	227.2＞199<br>227.2＞101	0.03	0.1
4-Hydroxybenzophenone (4-OH-BP)	1137-42-4	-	130	26/37	Negative	197＞120<br>197＞92	0.03	0.1
Benzophenone (BP)	119-61-9	3.18	97	13/28	Positive	183.2＞105<br>183.2＞77.3	0.06	0.02
BPA-d16	-	-	121	18/22	Negative	241.5＞223<br>241.5＞142	0.1	0.03
Cyproterone Acetate (CPA)	427-51-0	3.10	160	25/13	Positive	417＞279<br>417＞118.9	0.03	0.01

Gestodene<br>(GES)	60282-87-3	3.26	130	5/29	Positive	311＞108.9<br>311＞149	0.03	0.01
Norethindrone<br>(NTD)	68-22-4	2.97	125	21/29	Positive	299＞108.9<br>299＞119	0.01	0.004
Diethylstilbestrol<br>(DES)	6898-97-1	5.42	120	25	Negative	267.1＞221.8	0.05	0.006
Levonorgestrel<br>(LNG)	797-63-7	3.48	125	13/20	Positive	312.45＞299.4	0.01	0.004
Ethynyl Estradiol<br>(EE2)	57-63-6	3.67	125	9/18	Positive	295.4＞132.8<br>295.4＞159	0.01	0.004
Norethindrone-d16<br>(NTD-d16)	-	-	145	25/45	Positive	306.1＞134.1<br>306.1＞113	0.01	0.004

Table S2. Detailed georeferences of the specific sampling sites.

Sample site	Latitude (N)	Longitude (E)
H1	121.246005	31.393626
H2	121.357180	31.419326
H3	121.585154	31.349584
H4	121.534852	31.314612
H5	121.476947	31.279320
H6	121.410709	31.247439
H7	121.428896	31.185293
H8	121.401518	31.144931
H9	121.232267	31.046140
H10	121.227258	31.013524
H11	121.164213	30.898257
H12	121.513014	30.928158
H13	121.605111	31.031802
H14	121.649541	30.917133
W1	121.179179	31.383836
W2	121.283524	31.404648
W3	121.405522	31.469368
W4	121.509756	31.405070
W5	121.453881	31.351694
W6	121.492209	31.284817
W7	121.391855	31.294117
W8	121.624753	31.348783
W9	121.380172	31.127897

W10	121.273566	31.188779
W11	121.120206	31.136156
A1	121.363047	31.495153
A2	121.284454	31.430041
A3	121.216951	31.304633
A4	121.106938	31.240942
A5	121.156161	31.139963
A6	121.371619	30.957829
A7	121.392600	30.872183
A8	121.648587	30.855820
A9	121.708359	30.856941
L1	121.283886	31.486037
L2	121.066341	30.950691
L3	121.387476	30.958154
L4	121.201227	30.816296
L5	121.842592	31.058703
R1	120.972742	31.094031
R2	120.850678	30.909603
R3	121.241014	30.963861
R4	121.351556	30.979153
R5	121.424994	31.000236
R6	121.487197	31.020511
R7	121.469144	31.085361
R8	121.455492	31.142136
R9	121.485986	31.245114
R10	121.537239	31.256878

R11	121.551017	31.332717
R12	121.494933	31.372344
R13	121.508275	31.389822

Table S3. Recovery information of target compounds.

Target compounds	% Recovery (± SD)	% matrix Effect[a] (± SD)	% Accuracy[b]		
			0.5 (ng/mL)	5 (ng/mL)	50 (ng/mL)
BP-1	102.32±4.57	100.34±3.65	102	102	102
BP-2	93.45±9.21	89.54±4.27	98	102	102
BP-3	91.41±1.25	99.24±5.31	99	103	99
BP-4	91.23±7.65	94.31±4.67	102	100	100
2,3,4-OH-BP	102.34±4.95	89.31±1.35	103	99	100
BP	98.78±6.33	85.67±2.67	101	100	103
4-OH-BP	83.45±3.66	94.36±6.13	99	97	97
CPA	102.35±2.87	101.23±2.84	106	103	103
GES	99.89±4.14	103.54 ±2.13	101	105	105
NTD	87.94±3.03	88.75±1.56	100	99	100
DES	62.38±1.28	80.15±2.38	99	100	100
LNG	79.73±7.98	86.54±3.32	101	99	100
EE2	92.34±8.25	97.32±2.94	101	100	101

[a] In order to further evaluate the matrix effect, each of the native standard response was compared (n = 3, native standards were spiked after sample extraction) with the standards in Milli-Q water/MeOH (4:1, *v*/*v*). [b] Accuracy was calculated at three different concentrations for matrix spike with 10 repeat measurements, expressed as percent error of measured value to its nominal value.

Table S4. The concentrations of the target compounds in water samples and sediment samples.

	Metabolite	% DF[a]	*GM[b]	*AM[c]	*SD[d]	Min	Media	Max
**Wastewater (ng/mL)**	BP-1	84%	5.56	24.91	34.62	N.D.	5.85	131.25
	BP-2	82%	2.74	7.48	10.46	N.D.	5.02	52.81
	BP-3	95%	18.01	56.14	96.93	N.D.	15.5	400.53
	BP-4	71%	2.54	21.86	44.71	N.D.	3.91	188.12
	2,3,4-OH-BP	68%	1.88	14.24	26.59	N.D.	3.98	114.04
	BP	66%	6.22	68.48	86.64	N.D.	10.65	298.67
	4-OH-BP	66%	1.67	7.09	9.79	N.D.	3.55	38.56
	EE2	87%	7.59	29.82	69.50	N.D.	9.95	338.24
	LNG	71%	2.06	6.04	8.51	N.D.	2.31	39.37
	NTD	95%	6.09	12.23	18.93	N.D.	7.08	88.02
	DES	74%	2.59	6.19	9.60	N.D.	4.35	55.48
	GES	68%	1.51	4.68	10.83	N.D.	1.63	67.45
	CPA	82%	4.08	10.81	20.26	N.D.	7.41	119.46
**Sediment (ng/g dw)**	BP-1	74%	1.39	3.94	4.62	N.D.	1.61	17.02
	BP-2	53%	0.76	3.66	7.36	N.D.	1.63	45.85
	BP-3	92%	6.73	10.43	6.88	N.D.	9.55	30.21
	BP-4	87%	1.76	6.51	10.19	0.02	4.08	59.91
	2,3,4-OH-BP	76%	2.25	6.27	7.15	N.D.	3.61	33.42
	BP	76%	3.42	30.35	69.02	N.D.	5.81	356.92
	4-OH-BP	76%	1.81	5.71	7.07	N.D.	2.39	29.54
	EE2	97%	1.86	3.72	4.45	0.19	1.98	17.72
	LNG	71%	0.64	0.99	1.18	N.D.	0.61	5.83
	NTD	92%	2.01	3.84	4.26	N.D.	2.23	21.97
	DES	58%	0.67	1.12	1.16	N.D.	0.49	4.38

	GES	68%	0.96	2.06	2.41	N.D.	0.97	9.41
	CPA	79%	1.26	2.01	2.06	N.D.	1.45	11.18

Table S5. Toxicity data of BPs and SP from the ECOTOX database for the SSD model.

Target substance	Species	Endpoint	Endpoint value(mg/L)	Target substance	Species	Endpoint	Endpoint value(mg/L)
**BP-1**	Calanoid Copepod	EC50	1.50	**BP-4**	Haptophyte	EC50	10
	Calanoid Copepod	LC50	2.60		Mysid	EC50	10
	Flatworm	LC50	2.80		Sea Urchin, Echinoderm	EC50	10
	Goldfish	LOEC	0.48		Mediterranean Mussel	EC50	10
	Goldfish	NOEC	0.48		Flatworm	LC50	158
**BP-2**	Zebra Danio	EC50	4.70		Zebra Danio	LOEC	3
	Smooth Cauliflower Coral	EC50	0.019		Goldfish	LOEC	0.49
	Zebra Danio	LC50	11.81		Zebra Danio	NOEC	0.03
	Smooth Cauliflower Coral	LC50	28.315		Goldfish	NOEC	4.9
	Flatworm	LC50	8.30	**4-OH-BP**	Flatworm	LC50	15.8
	Fathead Minnow	LOEC	9.70	**2,3,4-OH-BP**	Flatworm	LC50	34.6
	Zebra Danio	LOEC	7.5	**BP**	Water Flea	EC50	0.28
	Fathead Minnow	LOEC	5		Fathead Minnow	EC50	13.7
	Goldfish	LOEC	4.78		Flatworm	LC50	15.8
	Smooth Cauliflower Coral	LOEC	0.0246		Bullfrog	LOEC	1.457774
	Dinoflagellate Order	LOEC	0.00246		Fathead Minnow	LOEC	6.38
	Zebra Danio	NOEC	3.693291		Duckweed	LOEC	1.5625
	Fathead Minnow	NOEC	5		Fathead Minnow	NOEC	3.31
	Goldfish	NOEC	4.78		Japanese Medaka	NOEC	1.822218
	Smooth Cauliflower Coral	NOEC	24.6	**CPA**	Calanoid Copepod	EC50	0.049
	Dinoflagellate Order	NOEC	0.00246		Scud	LC50	2.69117
**BP-3**	Haptophyte	EC50	0.01387		Water Flea	LOEC	2.1
	Mysid	EC50	0.71076		Fathead Minnow	LOEC	0.16
	Smooth Cauliflower Coral	EC50	0.436		Mediterranean Feather Star	LOEC	0.0003

Sea Urchin, Echinoderm	EC50	3.28		Atlantic Dogwinkle	LOEC	1.25
Mediterranean Mussel	EC50	3.47259		Netted Dog Whelk	LOEC	1.25
Smooth Cauliflower Coral	LC50	2.876		Great Pond Snail	LOEC	0.0287
Thin Finger Coral	LC50	0.036		Water Flea	NOEC	2.1
Smooth Cauliflower Coral	LC50	12.811		Calanoid Copepod	NOEC	0.032
Boulder Star Coral	LC50	0.074		Water Flea	NOEC	2.1
Mustard Hill Coral	LC50	0.34		Mummichog	NOEC	0.1
Great Star Coral	LC50	0.052		Fathead Minnow	NOEC	0.16
Coral	LC50	0.008		Tilapia	NOEC	100
Staghorn Coral	LC50	0.009		Mediterranean Feather Star	NOEC	0.003
Flatworm	LC50	0.9		Sea Urchin, Echinoderm	NOEC	0.003
Haptophyte	LOEC	0.3		Great Pond Snail	NOEC	0.0287
Mysid	LOEC	0.5	**NTD**	Water Flea	EC50	6.41
Japanese Medaka	LOEC	0.09		Fathead Minnow	LC50	0.0148
Zebra Danio	LOEC	0.11		Fathead Minnow	LOEC	0.00074
Japanese Medaka	LOEC	0.62		Channel Catfish	LOEC	0.0825
Zebra Danio	LOEC	1.04		Rainbow Trout	LOEC	0.1
Goldfish	LOEC	4.76		Water Flea	NOEC	500
Rainbow Trout	LOEC	0.749		Fathead Minnow	NOEC	0.0354
Midge	LOEC	0.91		Channel Catfish	NOEC	0.0825
Smooth Cauliflower Coral	LOEC	0		Rainbow Trout	NOEC	0.1
Sea Urchin, Echinoderm	LOEC	3.84	**LNG**	Clawed Frog	LOEC	0.000156
Mediterranean Mussel	LOEC	0.3		Clawed Frog	NOEC	0.000156
Haptophyte	NOEC	0.03	**EE2**	Zebra Danio	EC50	1.964171
Mysid	NOEC	0.375		Water Flea	LOEC	1
Goldfish	NOEC	4.76		Eastern Mosquitofish	LOEC	0.000106

Rainbow Trout	NOEC	0.08	Brown Trout	LOEC	0.296409
Midge	NOEC	0.91	Yellow Catfish	LOEC	0.001
Smooth Cauliflower Coral	NOEC	0.22	Brown Trout	LOEC	0.296409
Sea Urchin, Echinoderm	NOEC	1.92	Crimson-Spotted Rainbowfish	LOEC	0.000075
Mediterranean Mussel	NOEC	0.03	Atlantic Cod	LOEC	0.002964
			Eastern Mosquitofish	LOEC	0.000106
			Zebra Danio	LOEC	0.296409
			Brown Trout	LOEC	0.01925
			Sea Urchin, Echinoderm	LOEC	0.000005
			Mediterranean Mussel	LOEC	0.0005
